# Efficacy and Safety of Upadacitinib for Management of Moderate-to-Severe Atopic Dermatitis: An Evidence-Based Review

**DOI:** 10.3390/pharmaceutics14112452

**Published:** 2022-11-14

**Authors:** Yuliya Lytvyn, Asfandyar Mufti, Abrahim Abduelmula, Muskaan Sachdeva, Khalad Maliyar, Jorge R. Georgakopoulos, Jensen Yeung

**Affiliations:** 1 Temerty Faculty of Medicine, University of Toronto, Toronto, ON M5S 1A8, Canada; 2 Division of Dermatology, Department of Medicine, University of Toronto, Toronto, ON M5S 1A8, Canada; 3 Division of Dermatology, Department of Medicine, Sunnybrook Health Sciences Centre, Toronto, ON M4N 3M5, Canada; 4 Faculty of Medicine, Schulich School of Medicine and Dentistry, Western University, London, ON N6A 5C1, Canada; 5 Department of Dermatology, Women’s College Hospital, Toronto, ON M5S 1B2, Canada; 6 Probity Medical Research Inc., Waterloo, ON N2J 1C4, Canada

**Keywords:** atopic dermatitis, eczema, upadacitinib, JAK1 inhibitors

## Abstract

Atopic dermatitis (AD) is a common skin condition characterized by inflammation that presents with erythematous and pruritic skin. Its chronic relapse-remitting nature has a significant impact on the quality of life, and often requires ongoing management. Given the limited treatments available for AD, there remains a large need for effective and safe alternative therapies for long-term use. Janus kinase (JAK) inhibitors are a new class of agents that target the JAK-STAT pathway, which plays an important role in the production of proinflammatory cytokines involved in AD pathogenesis. Phase II and III clinical trials revealed that JAK inhibitors, such as upadacitinib, are effective and well-tolerated agents for the treatment of moderate-to-severe AD. As a result, upadacitinib was approved for use in patients with moderate-to-severe AD by the European Medicines Agency (2021), Health Canada (2021) and the FDA (2022) in the last year. It is important for dermatologists to be aware of the clinical evidence to continue incorporating the use of upadacitinib into the treatment algorithm for AD, which will ultimately lead to improved patient outcomes. Therefore, this review is an up-to-date summary of the clinical data available on the efficacy and safety of upadacitinib treatment for AD.

## 1. Introduction

Atopic dermatitis (AD) is a relapsing chronic inflammatory skin condition with 15–20% of people being affected in developed countries [1,2]. Skin manifestations of AD can occur prior to the age of 1 year in 60% of patients, and before 5 years in 90% of patients, while the onset of AD in adulthood is observed in 10% of patients [3]. AD is characterized by a dysfunction in the skin barrier, leading to recurrent pruritic, xerotic, and erythematous lesions. This dysfunction in the skin barrier also makes it more prone to antigen penetration and immune system dysregulation, which is associated with an increased risk of cutaneous infections [4,5]. The chronic relapse-remitting nature of AD through the patients’ lifetime significantly impacts the quality of life and often requires ongoing treatment [6,7,8]. The most commonly used treatment options include topical therapy, phototherapy, and systemic immunotherapy. Topical therapy is typically used to manage mild AD, while moderate-to-severe disease often requires systemic immunosuppressive agents, which are often associated with a number of potential side effects [7,9,10,11,12]. Up until a year ago (2021), dupilumab, an inhibitor of interleukin-4 (IL-4) receptor-α, was the only biologic agent available for the treatment of moderate-to-severe AD [13]. Despite the efficacy and safety demonstrated with the use of dupilumab in this patient population, only 40% of patients were shown to achieve clear or almost clear skin [14,15,16]. Moreover, dupilumab’s use may be limited by the associated adverse effects, such as conjunctivitis, reactions at the injection site, persistent head and neck erythema. Thus, AD treatment is challenging, and there is a need to increase available alternative targeted therapies that have a good long-term efficacy, tolerability, and safety profile. Over the last year, upadacitinib, a janus kinase (JAK) inhibitor, was approved for use in patients with moderate to severe AD by the European Medicines Agency (2021), Health Canada (2021) and the FDA (2022). It is important for clinicians to understand the data available for this new medication class to be able to treat patients with moderate-to-severe AD. The goal of this article is to provide an up-to-date thorough review of the evidence on the efficacy and safety of upadacitinib in the management of moderate-to-severe AD.

## 2. Methods

A search in the OVID PubMed, Medline, Google Scholar databases and on ClinicalTrials.gov was conducted up until 18 September 2022. The following keywords were used for the search: “upadacitinib,” “atopic dermatitis,” and “eczema.” Clinical trials and review articles were included in our summary of the literature. While all phase 3 clinical trials were presented in our narrative review, only one phase 2 trial with a large patient sample size was included. Studies reporting relevant data in human patients published in English language were included. The references of each identified article were reviewed for any studies that were not part of the original search.

## 3. The Role of Janus Kinase-Signal Transducer in the Pathogenesis of Atopic Dermatitis

The pathophysiology of AD is complex, involving an interplay of genetic predisposition, environmental and immunologic factors [6,7,17]. AD is characterized by a disruption in the epidermal skin barrier, hyperplasia of the epidermis and chronic inflammation associated with infiltration of T cells, eosinophils, and dendritic cell [18,19,20]. The epidermal disruption is partly a result of the decreased expression of structural proteins and lipids in response to an upregulation of interleukin (IL)-4, IL-13, and IL-33 [7,21,22]. AD is also associated with T cell activation, specifically T helper (Th)2 and Th22-centered inflammation in acute lesions [23] with some Th1 and Th17 components that are more prominent in the chronic disease stage [7,17,18,24,25,26]. Therefore, AD skin lesions were shown to have increased levels of cytokines, such as Th2 (IL-4, IL-13, IL-31), Th22 (IL-22), and Th1 (interferon [IFN]-γ) [27,28,29]. How much each of the pathways contributes to clinical presentation remains unclear and may potentially represent different molecular phenotypes of AD [23,24,26,30,31]. A multi-cytokine polarization of AD suggests that inhibiting more than one cytokine may achieve greater efficacy as a treatment strategy [31].

**Figure 1:**
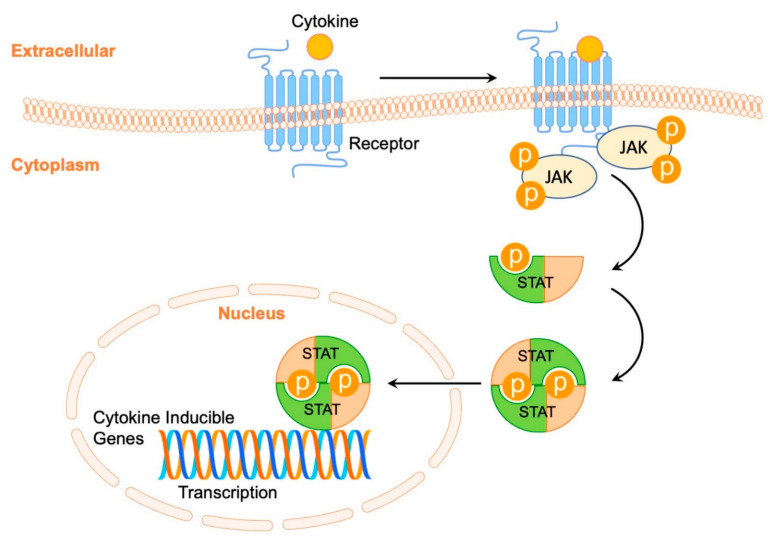
JAK-STAT pathway is a master regulator of immune function. Cytokines bind to their respective receptor, which cause the phosphorylation of JAK and activation of STAT proteins. Upon activation, STAT proteins dimerize, translocate into the nucleus, and upregulate transcription of proinflammatory cytokines and growth factors.

JAK-STAT pathway is an important regulator of the immune function. JAK activates STAT proteins, which work as transcription factors that translocate to the nucleus and upregulate a variety of growth factors and proinflammatory cytokines (Figure 1) [32]. The JAK kinase family consists of four molecules (JAK1, JAK2, JAK3 [33], and tyrosine kinase 2 [TYK2]). Similarly, there are six STAT proteins (STAT1, STAT2, STAT3, STAT4, STAT5, and STAT6) [34]. Each JAK protein is activated by different cytokines and can also activate distinct STAT proteins, creating different JAK-STAT combinations that determine a variety of features of immune cell development and function. IL-4 and IL-13 bind to the IL-4 receptor-α, and either to the γ chain or IL-13 receptor-α1 [35,36]. This results in activation of the JAK1/3, which, in turn, activates STAT6 [35,36]. The activation of STAT6 leads to increased expression of a proinflammatory extracellular matrix protein trophic to keratinocytes, namely periostin, which leads to the activation of JAK1/2 signalling pathways [37]. Additionally, IL-22 activates JAK1 and TYK2, leading to STAT3 phosphorylation, and is associated with disruption of the skin barrier, epidermal thickening, increased thymic stromal lymphopoietin and IL-33 expression [28,29]. IL-31 is implicated in JAK1/2 signaling by acting on keratinocytes and enhancing IL-24 release (JAK1/TYK2 pathway), leading to diminished filaggrin production, which is a major contributor to the disruption of the skin barrier [1,7]. Therefore, the dysregulation of JAK-STAT signaling has been reported to play a role in the underlying pathogenesis of multiple immune-mediated dermatoses, including but not limiting to AD, psoriatic arthritis, rheumatoid arthritis, and inflammatory bowel disease [38,39,40,41,42,43].

Given that JAK-STAT pathways regulate multiple steps known to be involved in the pathogenesis of AD, it is not surprising that all four JAK proteins were found to have enhanced signaling in AD [35]. Moreover, some preclinical research evidence suggests that neuronal JAK1 signaling is implicated in chronic itch [44]. Therefore, blocking JAK pathway signaling may reduce inflammation and itch associated with AD. Therapeutics that inhibit JAK-STAT pathways are an important focus of research, with a growing body of literature demonstrating that they are safe and efficacious for use in several inflammatory skin conditions, including AD [44]. The first generation of JAK inhibitors include tofacitinib, ruxolitinib, baricitinib and oclacitinib, however these lack specificity and have an increased risk of off-target effects, which may increase the concern regarding unwanted adverse effects [42]. As a result, a second generation of JAK inhibitors were developed that are selective for the JAK1 protein, namely abrocitinib and upadacitinib, which have both been approved by European Medicines Agency (2021 for both therapies), Health Canada (2022 for abrocitinib and 2021 for upadacitinib) and the FDA (2022 for both therapies) for use in moderate-to-severe atopic dermatitis. This literature review will be focused on upadacitinib, the first JAK1 inhibitor approved for use in AD.

## 4. Upadacitinib in Atopic Dermatitis

Upadacitinib (ABT-494, Rinvoq, Abbvie) is an oral selective inhibitor of JAK1 approved for use in moderate-to-severe rheumatoid arthritis in patients with intolerance or an inadequate response to methotrexate. Most recently (2021–2022), it was approved for use in moderate-to-severe AD in candidates for systemic therapy. Studies in healthy volunteers report that upadacitinib administration dose-dependently inhibits JAK1/JAK2-induced STAT3 phosphorylation and JAK1/JAK3-induced STAT5 phosphorylation. The inhibition of JAK1 by upadacitinib subsequently leads to decreased production of pro-inflammatory cytokines, specifically IL-6, IL-15, IFN-α, and IFN-γ. Upadacitinib is >40-fold more potent at JAK1 compared to JAK3 and 100-fold more potent at JAK1 compared to TYK2, with an IC50 of 43 nM for JAK1, 120 nM for JAK2, 2300 nM for JAK3, and 4700 nM for TYK2 [45]. Such high JAK1 selectivity may result in a better benefit-risk profile compared to less selective JAK inhibitors [46].

Upadacitinib is rapidly absorbed, with an oral availability of 76%, peak plasma concentrations at 1–2 h post-administration, a t_1/2_ of 4 h, and a mean terminal elimination half-life between 6 and 16 h [47,48,49]. A mild plasma-protein binding of 50% has been reported with this agent [47,48,49]. Approximately 20% of upadacitinib is eliminated unchanged in urine and 34% is excreted as metabolites, which are mainly by-products of cytochrome P450 (CYP) 3A4, with a minor contribution from CYP2D6 [47,48,49]. Therefore, the plasma concentration of upadacitinib is increased when it is co-administered with inhibitors of CYP3A4, such as ketoconazole, and plasma concentrations are decreased when co-administered with potent CYP3A4 inducers, such as rifampin [47,48,49].

## 5. Efficacy of Upadacitinib in Atopic Dermatitis Treatment

### 5.1. Phase II

A phase II double-blind, multicenter, placebo-controlled, parallel-group, dose ranging study (NCT02925117) examined the treatment effect of 7.5, 15, and 30 mg daily upadacitinib monotherapy compared to placebo in 167 patients with moderate-to-severe AD inadequately controlled by topical treatments [9]. After 16 weeks of treatment, there were significant benefits observed in all three treatment arms compared to placebo (Table 1) [9]. The primary endpoint reported was a mean reduction of 39%, 62% and 74%, respectively, in the Eczema Area and Severity Index (EASI) score compared to the 23% observed with placebo (*p* = 0.03, *p* < 0.001, and *p* < 0.001, respectively). There was a clear dose–response relationship, with the greatest clinical benefit in the 30 mg daily upadacitinib treatment group, where 50% of treated participants achieved EASI90 [49]. A significantly larger proportion of patients in all upadacitinib dosing groups achieved EASI75 compared to the 10% in the placebo group (7.5 mg upadacitinib: 29% of patients, *p* < 0.05; 15 mg upadacitinib: 52%, *p* < 0.001; 30 mg upadacitinib: 69%, *p* < 0.001) [49]. A 100% improvement in EASI was achieved in 2.4% (*p* = 0.43), 9.5% (*p* = 0.05), and 24% (*p* = 0.001) of patients in the 7.5 mg, 15 mg, and 30 mg upadacitinib treatment groups, respectively, compared to 0% in the placebo group [9]. Maximal EASI improvement was reported at week 4 and was maintained until week 16 [9]. EASI90 appeared to plateau between weeks 8 and 16, while EASI100 continued to increase at week 16 [9]. Investigator’s global assessment (IGA) was also recorded and shown to be superior in all upadacitinib treatment groups with an improvement of two grades or more from baseline along with improvements in patient-reported pruritus on the numerical rating scale (NRS) compared to placebo [1]. Clear or almost clear skin on the IGA scale was achieved by 14% (*p* < 0.05), 31% (*p* < 0.001), and 50% (*p* < 0.001) of patients treated with 7.5 mg, 15 mg, and 30 mg of daily upadacitinib [9]. Peak clearance was achieved and maintained by week 4 or 8 in all treatment groups. Improvement in patient reported pruritus was also dose-dependent and, most importantly, improvements in signs, symptoms and itch associated with AD were observed as early as 1 week of treatment in each of the treatment arms [9]. Such an early reduction in pruritus is important for patients with AD, as itching has a significant negative impact on patients’ quality of life. Pruritus improvement with JAK1 inhibition may be mediated by IL-31 inhibition or other factors that prompt itching in sensory neurons [44]. Reductions in AD body surface area (BSA) percentage were significantly greater in all groups treated with upadacitinib compared to placebo at each biweekly measurement, starting at week 2 [9]. Reductions in serum Th2 and Th22 biomarkers were observed as early as 2 weeks post-treatment with 15 mg and 30 mg of upadacitinib, suggesting that upadacitinib could have strong early effects on Th2 and Th22 axes that are characteristic of AD [9]. Clinical improvement was correlated with changes in absolute eosinophil count, CCL18, CCL6, and IL-22, while there were no trends with Th17 or IL-17 [9].

### 5.2. Phase III

MEASURE UP 1 and MEASURE UP 2 were replicate phase III, multicenter, randomized, placebo-controlled, double-blind, parallel group trials (NCT03569293 and NCT03607422, respectively) that evaluated the efficacy and safety of upadacitinib in patients (12 to 75 years) with moderate-to-severe AD that were candidates for systemic therapy [50]. A total of 1683 (MEASURE UP 1 *n* = 847 and MEASURE UP 2 *n* = 836) adolescent and adult patients were randomly assigned to receive daily 15 mg or 30 mg upadacitinib, or placebo. Similar to the phase II trial, in MEASURE UP 1 patients receiving upadacitinib 15 mg and 30 mg at 16 weeks achieved 70% and 80% EASI75, respectively, at week 16, compared to 16% in patients receiving placebo (*p* < 0.001, Table 1). In the MEASURE UP 2 patients receiving upadacitinib 15 mg and 30 mg at 16 weeks achieved 60% and 73% EASI75, respectively, at week 16, compared to 13% in patients receiving placebo (*p* < 0.001). Clear or almost clear skin was achieved by 48% and 62% of patients on upadacitinib, respectively, compared to 8% on placebo (*p* < 0.001), as measured by IGA [50]. All patients on upadacitinib experienced a clinically meaningful itch reduction as early as 1 day after first 30 mg upadacitinib dose (12% vs. 4% on placebo, *p* < 0.001) and 2 days after first 15 mg upadacitinib dose (16% vs. 3% on placebo, *p* < 0.001), which was maintained through week 16 [50].

The last published trial to date is a phase 3, double-blind, randomized AD UP study (NCT03568318), which evaluated the efficacy and safety of upadacitinib when combined to a topical corticosteroid over a 52-week time period in patients 12 to 75 years with moderate-to-severe AD that were candidates for systemic therapy [51,52]. A total of 901 patients were randomized to 15 mg or 30 mg upadacitinib or placebo in combination with a topical corticosteroid. Endpoints were assessed at 16 and 52 weeks of treatment. In this study, reported efficacy at 16 weeks was comparable to the previously reported Phase II and III trials, which was maintained through week 52 (Table 1) [51,52].

## 6. Safety of Upadacitinib in Atopic Dermatitis Treatment

The previous use of JAK inhibitors in conditions such as rheumatoid arthritis yielded safety reports that include risks of infection, malignancy, and venous thromboembolism (VTE). Clinical trials reporting on the use of upadacitinib, a selective JAK1 inhibitor, in patients with AD, have shown an acceptable and improved safety profile. The phase IIb trial in 166 patients 18 to 75 years old randomized to 7.5 mg, 15 mg, and 30 mg upadacitinib reported no deaths, no herpes zoster infections, no VTEs, and no malignancies [9]. Jaw pericoronitis and AD-worsening were the serious adverse events in the 7.5 mg upadacitinib group. Overall, 74%, 76%, and 79% of patients reported adverse events in the upadacitinib treatment groups, respectively, compared to 63% of patients in the placebo group (Table 2) [9]. The most common adverse effects, reported in ≥5% of treated patients, included upper respiratory tract infections, worsening of acne and AD, headache, nasopharyngitis and an asymptomatic increase in blood creatine phosphokinase. These were all mild or moderate in severity and not correlated with the treatment dose [9]. Although 38% of patients had a history of asthma, there were no asthma exacerbations reported on treatment [9]. Discontinuation of upadacitinib treatment due to adverse events was rare.

Some laboratory abnormalities were noted in a few patients with all upadacitinib dose groups in the phase II trials, specifically changes in blood bilirubin, alanine aminotransferase, and aspartate aminotransferase. These lab changes resolved with dosage adjustment or drug discontinuation [9]. A within-normal-range decrease in hemoglobin was noted with upadacitinib treatment [9]. There were no changes to absolute lymphocytes and a decrease in neutrophils was similar in the treatment and placebo arms.

There were no unexpected safety risks identified in the phase III trials. Under 3% of patients in each trial developed eczema herpeticum or herpes zoster while being treated with 15 mg or 30 mg upadacitinib [50,52]. Finally, there were no reported deaths, opportunistic infections, or VTEs in the phase III trials [50,52].

Overall, the most common adverse events associated with the use of upadacitinib were upper respiratory tract infections, worsening of AD, acne, laboratory abnormalities in hemoglobin, neutrophils, lipids, and creatine phosphokinase. The unexpected increase in acne occurrence was not considered severe; however, it may have further potential impacts on the quality of life of a patient that is already managing AD. The mechanism of acne onset with upadacitinib treatment is unclear and should be further investigated in future trials. The laboratory abnormalities in hemoglobin, neutrophils, lipids and creatine phosphokinase with upadacitinib use were also not considered clinically significant [54,55,56,57,58]. The neutropenia, anemia and thrombocytopenia are a result of the role JAK2 plays in erythropoietin and other colony-stimulating factors [38]. JAK1 blockade leads to the inhibition of IL-6 involved in hematologic genesis and may also contribute to partial JAK2 inhibitory effect [54,55,56,57,58]. Whether abnormalities in serum lipids occur because of a reaction to inflammation or as a consequence of the JAK1 inhibitor mechanisms is not known; however, this does require further investigation to determine the long-term cardiovascular risk of these agents. Importantly, there were no thromboembolic events reported in the upadacitinib trials, unlike non-specific JAK inhibitors, suggesting that selective JAK1 antagonism has an improved safety profile [59]. The side-effect profile with long-term use requires further investigation as we continue to use these agents over time in real-world clinical practice.

## 7. Upadacitinib Compared to Available Treatment Options for Treatment of Atopic Dermatitis

There are few treatment options available to patients with AD. The main alternative to upadacitinib at present is the subcutaneous injection of an IL-4Rα inhibitor, dupilumab [60]. A HEADS UP phase 3b trial was a multicenter, randomized, double-blind, double-dummy, active-controlled head-to-head comparison of the safety and efficacy of oral upadacitinib (30 mg once daily) with subcutaneous dupilumab (300 mg every other week) in 692 adults with moderate-to-severe AD who were candidates for systemic therapy [53]. After 16 weeks of treatment, 71.0% of patients on upadacitinib achieved EASI75, which was significantly greater than the 61.1% of patients achieving EASI75 on dupilumab (*p* = 0.006, Table 1) [53]. A greater proportion of patients achieved EASI75 by 1 week of treatment with upadacitinib compared to dupilumab (15.9% vs. 5.5%, respectively) and by week 4 (69.9% and 35.9%, respectively) [53]. Moreover, upadacitinib treated patients had significantly greater reductions in pruritus compared to dupilumab-treated patients as early as after 1 week of therapy (−31.4% vs. −8.8%, respectively) and this impact on pruritus was maintained at week 16 (−66.9% vs. 49.0%, respectively). The superior effectiveness of selective JAK1 inhibition may reflect the modulation of further cytokine pathways involved in the pathogenesis of AD, beyond the Th2 and Th22 cytokines impacted by dupilumab [61,62]. The safety profile also differed between these two therapies, where upadacitinib was most commonly associated with infections, herpes zoster, eczema herpeticum, and laboratory related adverse events compared to dupilumab, which was most commonly associated with conjunctivitis and injection site reactions [53]. Thus, each of these agents have distinct advantages, and having them both available to patients as treatment options allows for treatment to be personalized based on patient needs, preferences, responses or tolerance to treatment, contraindications, adverse effects, and cost. Some patients may prefer a subcutaneous injection of dupilumab that is perhaps less efficacious, with no laboratory monitoring, while other patients may find oral upadacitinib therapy more convenient, with faster and greater efficacy, accompanied by laboratory monitoring. Patients that are most impacted by pruritus may choose selective JAK1 inhibitors for a rapid, clinically significant improvement in pruritus. Studies are ongoing to investigate the efficacy and safety of upadacitinib in patients who did not respond to dupilumab treatment (Table 3).

Abrocitinib was the most recent JAK1 inhibitor approved for use in patients with moderate-to-severe AD. Abrocitinib 200 mg showed a similar efficacy to upadacitinib in both JADE MONO trials with at least 60% EASI75 over 12 weeks compared to 69% to 80% EASI 75 over 16 weeks with 30 mg upadacitinib [9,50,52,63]. Nevertheless, there are no data available from direct head-to-head trials to draw accurate conclusions on a comparison of these two JAK1-inhibitor agents. It is likely that real-world use studies or future head-to-head studies will determine clinically significant differences between the increasing options available for AD treatment. Ultimately, the availability of these two selective JAK1 inhibitors in the toolbox for the treatment of moderate-to-severe AD significantly broadened the treatment options available to patients suffering from this skin disease.

## 8. Conclusions

The need for more efficacious, safe, and long-term treatment options for moderate-to-severe AD has led to a strong motivation of producing novel agents to help patients suffering from this skin disease. The JAK-STAT signaling pathway plays an important role in regulating the immune system by targeting several inflammatory cytokines simultaneously, making it an ideal candidate for therapeutic intervention in a number of inflammatory conditions, including AD. The efficacy and safety of a selective JAK1 inhibitor, upadacitinib, were shown in a number of Phase II and III trials in adult and adolescent patients with AD. Subsequently, upadacitinib was the first in this class of medications to be approved for use in patients with moderate-to-severe AD by the European Medicines Agency (2021), Health Canada (2021) and the FDA (2022). These agents became a helpful addition to the toolbox of therapeutic options, with a rapid relief of pruritus, good oral bioavailability, and a good safety profile. Increasing therapeutic options for the treatment of moderate-to-severe AD offers an improved disease management that considers individual patient factors, preferences, and responses to treatment. Further long-term maintenance, efficacy and safety real-world and clinical studies are ongoing to continue evaluating how the use of upadacitinib can fill the unmet needs of patients with moderate-to-severe AD (Table 3).

## Figures and Tables

**Table 1:** Summary of efficacy outcomes for clinical trials completed with upadacitinib treatment in patients with moderate-to-severe atopic dermatitis.

Outcome	Medication and Dose	Guttman-Yassky et al. [9] (*n* = 167)	Measure Up 1 [50] (*n* = 847)	Measure Up 2 [50] (*n* = 836)	AD UP * [51,52] (*n* = 901)		Heads Up (*n* = 348) [53]
Duration of treatment		16 weeks	16 weeks	16 weeks	16 weeks	52 weeks	16 weeks
% of patients achieving EASI75	Upadacitinib 7.5 mg	29% done have SE	N/A	N/A	N/A	N/A	N/A
	Upadacitinib 15 mg	52%	70%	60%	65%	51%	
	Upadacitinib 30 mg	69%	80%	73%	77%	69%	71%
	Placebo	10%	16%	13%	26%	N/A	N/A
	Dupilumab 300 mg every other week	N/A	N/A	N/A	N/A	N/A	61%
% of patients achieving EASI90	Upadacitinib 7.5 mg	14%	N/A	N/A	N/A	N/A	N/A
	Upadacitinib 15 mg	26%	53%	42%	43%	38%	
	Upadacitinib 30 mg	50%	65.8%	59%	63%	55%	61%
	Placebo	2%	8.1%	5%	13%	N/A	
	Dupilumab 300 mg every other week	N/A	N/A	N/A	N/A	N/A	39%
% of patients achieving EASI100	Upadacitinib 7.5 mg	2.4%	N/A	N/A	N/A	N/A	N/A
	Upadacitinib 15 mg	9.5%	17%	14%	12%	13%	
	Upadacitinib 30 mg	24%	27%	19%	23%	24%	28%
	Placebo	0%	2%	0.7%	1.3%	N/A	N/A
	Dupilumab 300 mg every other week	N/A	N/A	N/A	N/A	N/A	8%
% improvement EASI	Upadacitinib 7.5 mg	39%	N/A	N/A	N/A	N/A	N/A
	Upadacitinib 15 mg	62%	80%	74%	78%	68%	N/A
	Upadacitinib 30 mg	74%	88%	85%	87%	77%	N/A
	Placebo	23%	41%	35%	46%	N/A	N/A
	Dupilumab 300 mg every other week	N/A	N/A	N/A	N/A	N/A	N/A
% of patients achieving IGA 0/1	Upadacitinib 7.5 mg	14%	N/A	N/A	N/A	N/A	N/A
	Upadacitinib 15 mg	31%	48%	39%	40%	34%	N/A
	Upadacitinib 30 mg	50%	62%	52%	59%	45%	N/A
	Placebo	3%	8%	5%	11%	N/A	N/A
	Dupilumab 300 mg every other week	N/A	N/A	N/A	N/A	N/A	N/A
% of patients who achieved worst pruritus NRS improvement ≥ 4	Upadacitinib 7.5 mg	24%	N/A	N/A	N/A	N/A	N/A
	Upadacitinib 15 mg	59%	60%	60%	51%	45%	
	Upadacitinib 30 mg	53%	52%	42%	64%	58%	55%
	Placebo	6%	12%	9%	15%	N/A	N/A
	Dupilumab 300 mg every other week	N/A	N/A	N/A	N/A	N/A	36%

EASI: Eczema Area and Severity Index; IGA: Investigator’s global assessment; N/A: not applicable; NRS: numerical rating scale. * All treatment groups had topical corticosteroids co-administered.

**Table 2:** Summary of safety outcomes for clinical trials completed with upadacitinib treatment in patients with moderate-to-severe atopic dermatitis.

Adverse Event	Medication and Dose	Guttman-Yassky et al. [9] (*n* = 167) 16 Weeks	Measure Up 1 [50] (*n* = 847) 16 Weeks	Measure Up 2 [50] (*n* = 836) 16 Weeks	AD UP * [51,52] (*n* = 901) 16 Weeks	AD UP * [51,52] (*n* = 901) 52 Weeks
Any AE, *n* (%)	Upadacitinib 7.5 mg	31(74)	N/A	N/A	N/A	N/A
	Upadacitinib 15 mg	32 (76)	176 (63)	166 (60)	200 (67)	1730 (338)
	Upadacitinib 30 mg	33 (79)	209 (73)	173 (61)	215 (72)	1848 (347)
	Placebo	25 (63)	166 (59)	146 (53)	190 (63)	N/A
Serious AE, *n* (%)	Upadacitinib 7.5 mg	2 (4.8)	N/A	N/A	N/A	N/A
	Upadacitinib 15 mg	1 (2.4)	6 (2)	5 (2)	7 (2)	41 (8)
	Upadacitinib 30 mg	0 (0)	8 (3)	7 (3)	4 (1)	43 (8)
	Placebo	1 (2.5)	8 (3)	8 (3)	9 (3)	N/A
AE leading to drug discontinuation, *n* (%)	Upadacitinib 7.5 mg	4 (9.5)	N/A	N/A	N/A	N/A
	Upadacitinib 15 mg	2 (4.8)	4 (1)	11 (4)	4 (1)	20 (3.9)
	Upadacitinib 30 mg	4 (9.5)	11 (4)	7 (3)	4 (1)	20 (3.8)
	Placebo	3 (7.5)	12 (4)	12 (4)	7 (2)	N/A
Infection, *n* (%)	Upadacitinib 7.5 mg	22 (52)	N/A	N/A	N/A	N/A
	Upadacitinib 15 mg	18 (43)	5 (2)	9 (3)	6 (2)	28 (5.5)
	Upadacitinib 30 mg	17 (41)	9 (3)	0 (0)	9 (3)	51 (9.6)
	Placebo	8 (20)	4 (1)	0 (0)	3 (1)	N/A
Serious Infection, *n* (%)	Upadacitinib 7.5 mg	2 (4.8)	N/A	N/A	N/A	N/A
	Upadacitinib 15 mg	1 (2.4)	2 (1)	1 ( < 1)	3 (1)	14 (2.7)
	Upadacitinib 30 mg	0 (0)	2 (1)	2 (1)	0 (0)	12 (2.3)
	Placebo	0 (0)	0 (0)	2 (1)	3 (1)	N/A
Hepatic Disorder, *n* (%)	Upadacitinib 7.5 mg	0 (0)	N/A	N/A	N/A	N/A
	Upadacitinib 15 mg	2 (4.8)	5 (2)	2 (1)	6 (2)	41 (8)
	Upadacitinib 30 mg	0 (0)	8 (3)	4 (1)	3 (1)	26 (5)
	Placebo	1 (2.5)	2 (1)	4 (1)	5 (2)	N/A
Anemia, *n* (%)	Upadacitinib 7.5 mg	0 (0)	N/A	N/A	N/A	N/A
	Upadacitinib 15 mg	0 (0)	1 ( < 1)	2 (1)	0 (0)	7 (1.4)
	Upadacitinib 30 mg	1 (2.4)	8 (3)	4 (1)	3 (1)	13 (2.4)
	Placebo	0 (0)	2 (1)	4 (1)	1 (0.3)	N/A
Neutropenia, *n* (%)	Upadacitinib 7.5 mg	1 (2.4)	N/A	N/A	N/A	N/A
	Upadacitinib 15 mg	2 (4.8)	4 (1)	2 (1)	2 (1)	10 (2)
	Upadacitinib 30 mg	2 (4.8)	8 (3)	4 (1)	3 (1)	15 (2.8)
	Placebo	0 (0)	2 (1)	4 (1)	0 (0)	N/A
Lymphopenia, *n* (%)	Upadacitinib 7.5 mg	0 (0)	N/A	N/A	N/A	N/A
	Upadacitinib 15 mg	1 (2.4)	1 ( < 1)	0 (0)	0 (0)	2 (0.4)
	Upadacitinib 30 mg	0 (0)	2 (1)	1 ( < 1)	0 (0)	1 (0.2)
	Placebo	0 (0)	2 (1)	0 (0)	1 (0.3)	N/A
AE in ≥5% of patients in any group		N/A	N/A	N/A	N/A	N/A
URTI, *n* (%)	Upadacitinib 7.5 mg	7 (17)	N/A	N/A	N/A	N/A
	Upadacitinib 15 mg	5 (12)	25 (9)	19 (7)	21 (7)	45 (10.2)
	Upadacitinib 30 mg	5 (12)	38 (13)	17 (6)	23 (8)	45 (10.3)
	Placebo	4 (10)	20 (7)	12 (4)	22 (7)	N/A
AD worsening, *n* (%)	Upadacitinib 7.5 mg	6 (14)	N/A	N/A	N/A	N/A
	Upadacitinib 15 mg	2 (4.8)	9 (3)	8 (3)	11 (4)	47 (11)
	Upadacitinib 30 mg	4 (9.5)	4 (1)	4 (1)	2 (1)	29 (7)
	Placebo	2 (5.0)	26 (9)	26 (9)	20 (7)	N/A
Acne, *n* (%)	Upadacitinib 7.5 mg	4 (9.5)	N/A	N/A	N/A	N/A
	Upadacitinib 15 mg	2 (4.8)	19 (7)	35 (13)	30 (10)	62 (14)
	Upadacitinib 30 mg	6 (14)	49 (17)	41 (15)	41 (14)	81 (19)
	Placebo	1 (2.5)	6 (2)	6 (2)	6 (2)	N/A
Headache, *n* (%)	Upadacitinib 7.5 mg	3 (7.1)	N/A	N/A	N/A	N/A
	Upadacitinib 15 mg	3 (7.1)	14 (5)	18 (7)	15 (5)	29 (7)
	Upadacitinib 30 mg	4 (9.5)	19 (7)	20 (7)	14 (5)	28 (6)
	Placebo	1 (2.5)	12 (4)	11 (4)	15 (5)	N/A
Nasopharyngitis, *n* (%)	Upadacitinib 7.5 mg	2 (4.8)	N/A	N/A	N/A	N/A
	Upadacitinib 15 mg	4 (9.5)	22 (8)	16 (6)	37 (12)	76 (17)
	Upadacitinib 30 mg	3 (7.1)	33 (12)	18 (6)	40 (13)	73 (17)
	Placebo	1 (2.5)	16 (6)	13 (5)	34 (11)	N/A
Blood CPK increased, *n* (%)	Upadacitinib 7.5 mg	0 (0)	N/A N/A	N/A	N/A	N/A
	Upadacitinib 15 mg	3 (7.1)	16 (6)	9 (3)	13 (4)	37 (8)
	Upadacitinib 30 mg	4 (9.5)	16 (6)	12 (4)	18 (6)	49 (11)
	Placebo	2 (5.0)	7 (3)	5 (2)	7 (2)	

AD: atopic dermatitis; AE: adverse event; CPK: creatine phosphokinase; N/A: not applicable; URTI: upper respiratory tract infection. * All treatment groups had topical corticosteroids co-administered.

**Table 3:** Ongoing clinical trials of upadacitinib treatment in patients with moderate-to-severe atopic dermatitis.

ClinicalTrials.gov	Phase	Patient Population	Interventions (Duration)	Primary Outcome
**NCT03646604**	I	Pediatric [articipants (6–12 years) with severe AD (*n* = 32)	Upadacitinib (one dose)	Cmax Tmax AUCtau Oral Clearance Number of participants with treatment emergent adverse events
**NCT03661138** (Rising Up)	III	Adolescents and adults (12–75 years) with moderate to severe atopic dermatitis (*n* = 272)	Upadacitinib or Placebo in Combination With Topical Corticosteroids (141 weeks)	Number of participants experiencing adverse events
**NCT04195698**	III	Adults (18–75 years) with moderate to severe AD, successfully completed treatment with either Dupilumab or Upadacitinib (*n* = 485)	Upadacitinib (52 weeks)	Number of participants with adverse events
**NCT05507580** (Flex-Up)	IIIb/IV	Adults (18–64 years) with moderate to severe AD (*n* = 600)	Upadacitinib (12 weeks, 24 weeks)	Percentage of participants achieving EASI 75 Percentage of participants achieving EASI 90 Percentage of participants achieving EASI 90 and WP-NRS of 0 or 1
**NCT05394792** (CAN UpTIMISE)	Observational Prospective	Adults with moderate-to-severe AD, inadequate responce or discontinuation of dupilumab (*n* = 100)	Upadacitinib (up to 4 months)	Percentage of participants achieving validated investigator Global assessment for atopic dermatitis vlGA-AD of 0 or 1
**NCT05139836** (UP-TAINED)	Observational Prospective	Adults with AD (*n* = 772)	Upadacitinib (up to 24 months)	Percentage of participants achieving disease control Defined by ADCT total score <7 points Percentage of participants achieving disease control Defined by ADCT total score <7 points among participants Who achieved disease control at month 3
**NCT05081557** (AD-VISE)	Observational Prospective	Adults and adolescents (≥12 Years Old) with AD 9 *n* = 975)	Upadacitinib (up to 24 months)	Upadacitinib utilization patterns vIGA-AD 0/1 vIGA-AD 0/1 among participants who achieved vIGA-AD 0/1 at Month 4
**NCT05029895**	Observational Prospective	Adolescents (≥12–18 Years) with AD (*n* = 170)	Upadacitinib (2 years)	Percentage of participants with serious infection
**NCT05451316** (ADMIRE)	Observational Prospective	Adolescents and adults (≥12) with moderate to severe prurigo-type AD (*n* = 200)	Upadacitinib (up to 12 weeks)	Percentage of participants achieving WP-NRS reduction ≥ 4

AD: Atopics Dermatitis; ADCT: Atopic Dermatitis Control Tool; AUCtau: Area under the plasma concentration-time curve within a dosing interval; Cmax: Maximum Plasma Concentration; Tmax: Time to Maximum Observed Plasma Concentration; vlGA-AD: Validated Investigator Global Assessment for Atopic Dermatitis; WP-NRS: Worst Pruritus Numerical Rating Scale.

## Data Availability

This was a review study and thus, there was no data collection performed.
